# Septic Systems Contribution to Phosphorus in Shallow Groundwater: Field-Scale Studies Using Conventional Drainfield Designs

**DOI:** 10.1371/journal.pone.0170304

**Published:** 2017-01-20

**Authors:** Sara Mechtensimer, Gurpal S. Toor

**Affiliations:** Soil and Water Quality Laboratory, Gulf Coast Research and Education Center, Institute of Food and Agricultural Sciences, University of Florida, Wimauma, Florida, United States of America; National Sun Yat-sen University, TAIWAN

## Abstract

Septic systems can be a potential source of phosphorus (P) in groundwater and contribute to eutrophication in aquatic systems. Our objective was to investigate P transport from two conventional septic systems (drip dispersal and gravel trench) to shallow groundwater. Two new *in-situ* drainfields (6.1 m long by 0.61 m wide) with a 3.72 m^2^ infiltrative surface were constructed. The drip dispersal drainfield was constructed by placing 30.5 cm commercial sand on top of natural soil and the gravel trench drainfield was constructed by placing 30.5 cm of gravel on top of 30.5 cm commercial sand and natural soil. Suction cup lysimeters were installed in the drainfields (at 30.5, 61, 106.7 cm below infiltrative surface) and piezometers were installed in the groundwater (>300 cm below infiltrative surface) to capture P dynamics from the continuum of unsaturated to saturated zones in the septic systems. Septic tank effluent (STE), soil-water, and groundwater samples were collected for 64 events (May 2012–Dec 2013) at 2 to 3 days (n = 13), weekly (n = 29), biweekly (n = 17), and monthly (n = 5) intervals. One piezometer was installed up-gradient of the drainfields to monitor background groundwater (n = 15). Samples were analyzed for total P (TP), orthophosphate-P (PO_4_–P), and other–P (TP—PO_4_-P). The gravel trench drainfield removed significantly (p<0.0001) greater TP (~20%) than the drip dispersal in the first 30.5 cm of the drainfield. However, when STE reached >300 cm in the groundwater, both systems had similar TP reductions of >97%. After 18 months of STE application, there was no significant increase in groundwater TP concentrations in both systems. We conclude that both drainfield designs are effective at reducing P transport to shallow groundwater.

## Introduction

Septic systems can effectively treat wastewater when correctly sited, operated, and maintained. However, there is increasing evidence of phosphorus (P) transport from septic systems located in areas with sandy soil and high groundwater tables [1, 2]. Previous research links nutrient rich groundwater discharges and eutrophication in receiving water bodies [3–5] making managing nutrient sources to groundwater an important component of overall aquatic health. Approximately 25% of the U.S. population and 31% of Florida population rely on septic systems to treat and dispose household wastewater [6]. These systems are frequently utilized in rural or rapidly developing urban/suburban areas where centralized treatment facilities are too costly or cannot keep pace with rapid urban development.

In the U.S., the two most common conventional drainfield designs are the drip dispersal and gravel trench systems. The gravel trench system utilizes a gravel layer below the drip line, allowing the sides and bottom of the trench to act as infiltrative surfaces. The drip dispersal system omits the gravel layer and utilizes only the bottom of the bed as the infiltrative surface [7]. Although the drip dispersal system is the most common design due to the reduced cost and land area needed for treatment, gravel trenches are generally more desirable than drip dispersal systems as they provide up to five-times more side-wall area for infiltration than beds [7].

Phosphorus attenuation in septic system drainfields utilizes a combination of biotic and abiotic processes including sorption/precipitation reactions, plant uptake, and mineralization/ immobilization by microbes [8, 9]. Researchers agree the dominant P attenuation mechanisms in drainfields are sorption/ precipitation reactions. Phosphorus attenuation can occur throughout the drainfield, but researchers have observed rapid attenuation within close proximity (1–3 m) of the infiltration pipes [10] due to the reduction/oxidation (redox) changes resulting in precipitation of P minerals [11]. Wilhelm et al. [8] found that septic tank effluent (STE) oxidation and the soils buffering capacity influenced the pH and redox potential in the drainfield, which in turn, affected the P species, solubility, and charge of cations (Al, Fe, Ca, and Mg) associated with P minerals, effectively controlling whether P will remain mobile in the drainfield ([9]. For example, extensive monitoring of a drainfield located in a non-calcareous soil (87% sand, 13% silt) with groundwater depth from 1.75 to 2.60 m found that the oxidation of STE resulted in lower pH and orthophosphate (PO_4_–P) concentrations of <0.01 mg L^–1^ throughout 13 year of operation [10, 12]. In contrast, monitoring of a drainfield located in calcareous soil (97% sand, 2.8% silt and clay) with groundwater depth of ~2.25 m found a plume with PO_4_–P concentrations of 4.8 mg L^–1^ (equivalent to 75% of STE concentration) advancing 1 m per year from the drainfield after 17 year of operation [10]. These examples illustrate how redox conditions, pH, and soil characteristics (buffering capacity) influence P transport from the drainfield to groundwater.

Limited research has been conducted to investigate P dynamics in conventional drainfields in areas with sandy soils and shallow groundwater. The objectives of this study were to (1) investigate the P dynamics in the two conventional drainfield types in areas with shallow water tables and sandy soils, (2) identify which P forms are more mobile in the drainfields, and (3) determine if seasonality (wet or dry) play a role in P transport to shallow groundwater.

## Materials and Methods

### Study Site

The study site was located at the Gulf Coast Research and Education Center of the University of Florida in Wimauma, Florida, USA. At the site, the soil is loamy sand and is classified as a Spodosol, zolfo fine series (sandy siliceous, Hyperthermic Oxyaquic Alorthods). During the study period (May 2012 to Dec 2013), the mean monthly wet season temperature (June–September) was 25°C. Total annual rainfall during 2012–2013 was 115–131 cm, with mean monthly rainfall ranging from 0.3 to 41.9 cm [13]. During 2012 and 2013 wet seasons (June to September), total rainfall was 86–96 cm (73–75% of total annual rainfall), which was higher than the 10 year average wet season rainfall (2004–2013) of 76 cm (65% of total annual rainfall).

### Construction and Instrumentation of Septic System Drainfields

Two *insitu* drainfields (drip dispersal and gravel trench) were constructed approximately 10 m apart to avoid any potential plume interactions. Each drainfield was 6.1 m long by 0.61 m wide (3.72 m^2^ infiltrative surface), with an approximate horizontal and vertical slope ratio of 2:1. The drainfields shared two concrete septic tanks (9,500 and 4,750 L) that received effluent from graduate housing units and business units of Gulf Coast Research and Education Center (~50 employees) of the University of Florida. The effluent from these tanks was directed to a dosing tank that monitored the daily flow to the drainfields with a flow meter.

The drip dispersal system was constructed by placing 30.5 cm of commercial sand on top of natural soil, whereas the gravel trench system had an additional 30.5 cm of gravel on top of the sand layer. Table 1 lists basic properties of soil and commercial sand used in the construction of drainfields.

**Table 1:** Physiochemical properties of drainfield sand and soil (mean±standard deviation). Data for soil texture, pH, and EC from De and Toor [14].

Parameters	Natural Soil (n = 5)	Commercial Sand (n = 5)
Sand (%)	86.5±1.4	100±0
Silt (%)	8.5±1.4	0
Clay (%)	5.0±1.8	0
Texture	Loamy sand	Commercial sand
pH	6.4±0.09	7.2±0.13
EC (dS m^-1^)	0.06±0.01	0.06±0.01
Water-soluble P (mg kg^-1^)	4.99±0.63	4.92±0.37
Total Ca (mg kg^-1^)	993±129.8	734.8±58.8
Total Mg (mg kg^-1^)	122.5±14.5	41.8±14.4

**Fig 1:**
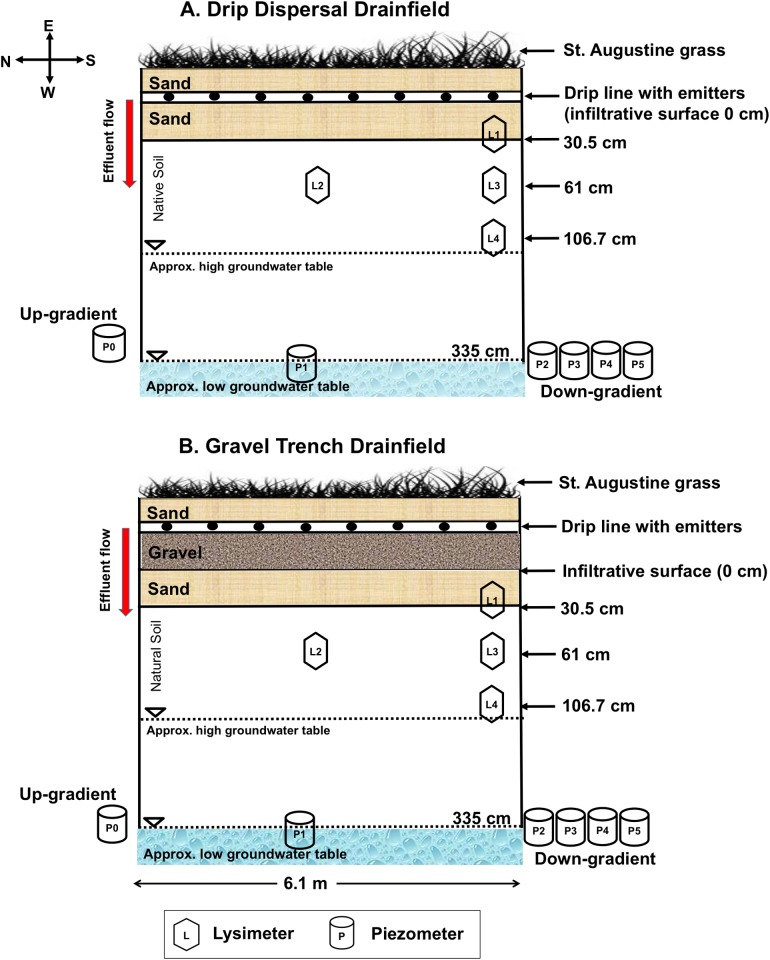
A north-south longitudinal cross-section (not to scale) of the drainfields showing vadose zone and groundwater monitoring instruments: (A) drip dispersal and (B) gravel trench systems.

In both systems, a drip line was placed on top of the drainfield and covered with 15 cm of commercial sand before planting St. Augustine grass (*Stenotaphrum secundatum)* (Fig 1). Two rows of pressure-dosed drip line with a total of 40 emitters (30.5 cm apart) were placed on top of the sand (drip dispersal) and gravel (gravel trench) to ensure uniform distribution of effluent. The drainfields received 120 L day^–1^ of STE, equivalent to the maximum allowable rate of 32 L m^–2^ day^–1^ for Florida loamy sand soils in 6-doses (20 L dose^–1^; 0.5 L per emitter^–1^ per dose^–1^) at 4 hr intervals [15].

Each drainfield was instrumented with a total of four suction cup lysimeters (5.1 cm diameter; Soil Moisture Equipment Corporation, Santa Barbara, CA). One suction lysimeter (L2) was located in the center of the drainfield at a depth of 61 cm below infiltrative surface with the remaining lysimeters were placed at the south end of the drainfield at a depth of 30.5 cm (L1), 61 cm (L3) and 106.7 cm (L4) (Fig 1). Each drainfield had a total of five 2.54 cm diameter standpipe piezometers (Geokon Inc., Lebanon, NH), with a 12.7 cm PVC screen interval and a 70 micron pore diameter, located at 3.1–3.4 m below the infiltrative surface. Of these, two piezometers located in the center (P1) and south end of the drainfield (P2) were monitored from events 1 to 48 (5/4/12–3/14/13), whereas three piezometers (P3–P5) were monitored from events 49 to 64 (3/28/13–12/12/13). One piezometer was installed up-gradient of the drainfields to monitor background groundwater. All subsurface monitoring devices were sealed with a bentonite seal above the ceramic cup to reduce preferential flow from the surface.

### Sample Collection and Processing

A total of 64 sampling events were conducted from May 2012 to Dec 2013. Initially, STE, soil-water, and groundwater were collected every 2 to 3 days (n = 13), then at weekly (n = 29), biweekly (n = 17), and finally at monthly (n = 5) intervals. This sampling regime was used to capture the temporal changes in treatment performance throughout the study period. Background groundwater samples were collected over 15 sampling events from April 2013 to Dec 2013 (n = 12 biweekly, n = 3 monthly) from a piezometer installed up-gradient of the drainfields. Soil-water samples from each suction cup lysimeter were collected after applying 50 kPa of vacuum pressure for 48 hr using a peristaltic pump. Groundwater samples were collected from the piezometers following purging of three equipment volumes following Florida Department of Environmental Protection standard operation procedures [16]). All samples (STE, soil-water, and groundwater) were placed in 250 mL bottles and transported on ice to the lab. Sub-samples were filtered using 0.45 μm filter paper (Pall Life Sciences, Pall Corporation, Ann Arbor, MI, USA), acidified with sulfuric acid, and stored at 4°C until analysis.

Unfiltered and filtered samples (STE, soil-water, and groundwater) were analyzed for total P (TP) and PO_4_–P, respectively, on a Seal AA3 Auto Analyzer (Seal Analytical, Mequon, WI, USA) using US EPA Method 365.1. For TP analysis, unfiltered samples were first digested using persulfate [17], which converts organic P and polyphosphates into PO_4_. Other–P was calculated as the difference between TP and PO_4_–P and consists of dissolved unreactive P (DUP), particulate reactive P (PRP), and particulate unreactive P (PUP), according to the physiochemical fractionation scheme [18].

### Statistical Analysis

Due to similar measurement results in the center and south end of the drainfields, mean data from groundwater monitoring instruments P1–P2 from 5/4/12 to 3/14/13 (events 1–48) are presented along with P3–P5 from 3/28/13 to 12/12/13 (events 49–64) as a combined series of 64 events and represented as P1–P5 (>300 cm below the infiltrative surface). Similar data were observed for lysimeters installed 61 cm in the center and south end of the drainfield, thus, data from L2 and L3 are combined and hereafter presented as L2–L3 (61 cm). Mean, median, and range were calculated in Microsoft Excel 2007.

A two-way ANOVA was conducted in JMP Pro 11 [19] to test the difference between P concentrations (n = 64) in the drip dispersal and gravel trench systems with depth (system/depth) and with season (wet n = 26; dry n = 38) (Season). The first factor in the two-way ANOVA was system/depth with a total of 10 variables. The variables included STE, background groundwater concentration, and two systems (drip dispersal and gravel trench) with four depths of 30.5 cm, 61 cm, 106.7 cm, and >300 cm below the infiltrative surface. By using this design, the two-way ANOVA only determined whether a difference occurred between any of 10 system/depth variables. To determine where the difference occurred, we used a Tukey table, which links similar values together with a letter and the values not linked are significantly different. The second factor in the two-way ANOVA was season, which determined the difference between P concentrations in the wet (June–September) and dry (October–May) season. A full factorial was used to evaluate the interaction between the drainfield systems with depth (system/depth) and season (wet/dry). The full factorial had 20 variables (2-seasons, 10-system/depth) and provided insights into any seasonal difference between the systems at any depth.

## Results

### Phosphorus Dynamics From Drainfields to Groundwater

**Fig 2:**
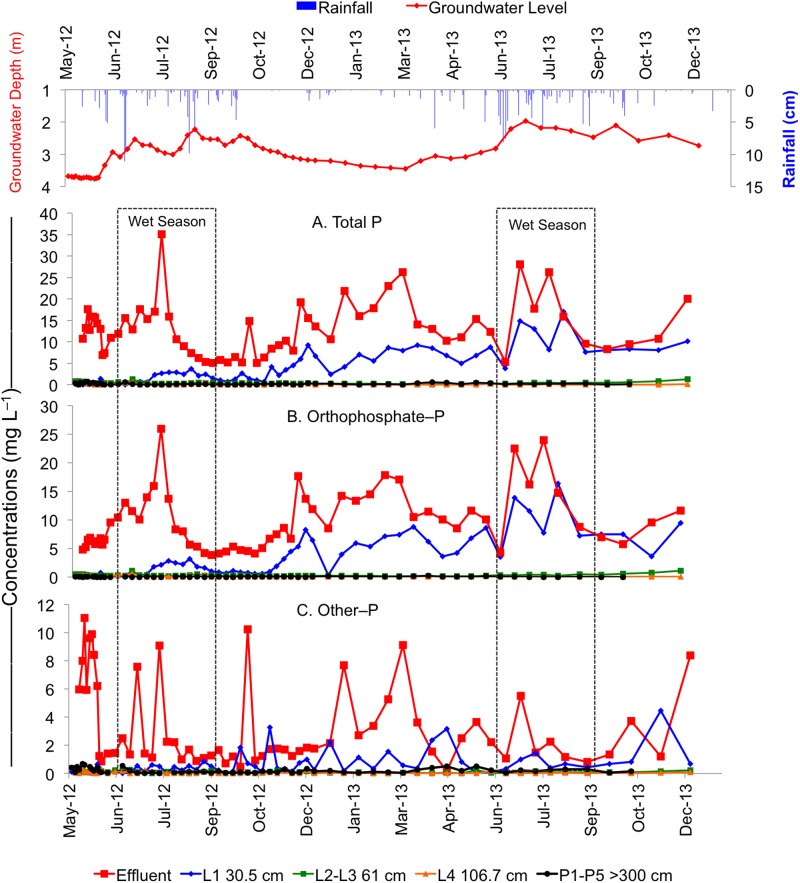
Temporal variability in concentrations of (A) total P, (B) PO_4_–P, and (C) other–P in the effluent, unsaturated zone lysimeters located at 30.5 cm (L1), 61 cm (average of L2 and L3), and 106.7 cm (L4) below the infiltrative surface, and saturated zone piezometers (average of P1 to P5) located at >300 cm depth in the groundwater in the drip dispersal septic system.

**Fig 3:**
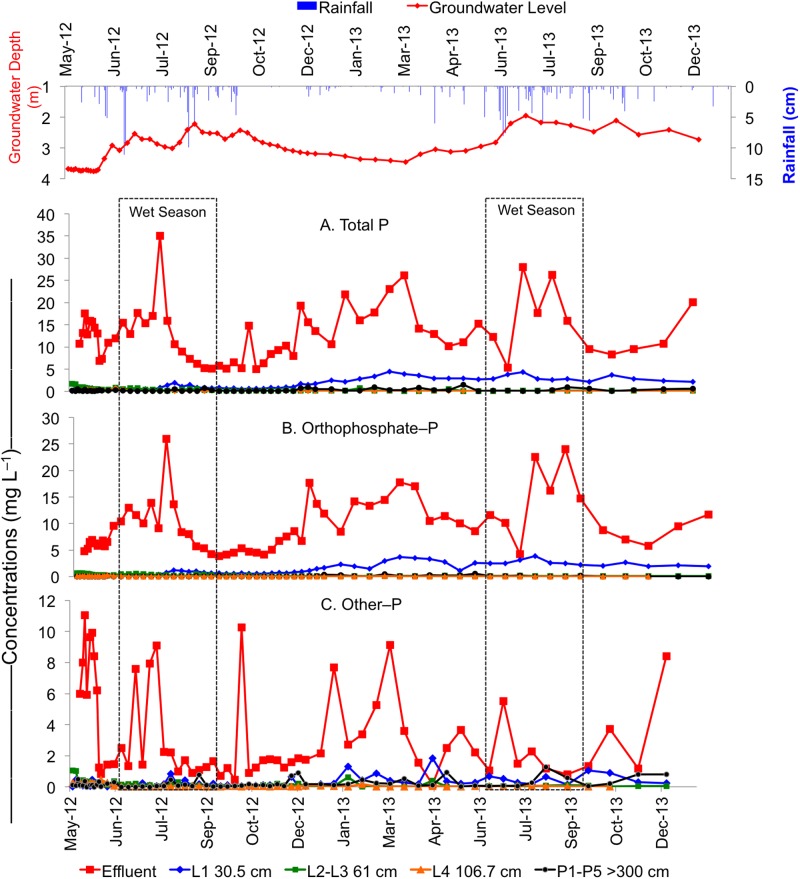
Temporal variability in concentrations of (A) total P, (B) PO_4_–P, and (C) other–P in the effluent, unsaturated zone lysimeters located at 30.5 cm (L1), 61 cm (average of L2 and L3), and 106.7 cm (L4) below the infiltrative surface, and saturated zone piezometers (average of P1 to P5) located at >300 cm depth in the groundwater in the gravel trench system.

Concentrations of TP, PO_4_–P, and other–P significantly decreased from STE to 30.5 cm depth below the infiltrative surface in both the drip dispersal and gravel trench drainfields (Table 2, Figs 2 & 3). In the drip dispersal drainfield, the elevated PO_4_–P and TP concentrations from July 2013 to Dec 2013 and the maximum concentration of other–P observed in the second dry season (Fig 2) are evidence of STE build up at 30.5 cm depth, indicating that the system was not at equilibrium and P was applied faster than it could be sorbed. In contrast, gravel trench drainfield did not show any marked increase in both P forms over the study period (Fig 3). When comparing the two drainfields, the gravel trench had significantly less mean PO_4_–P concentrations at 30.5 cm (1.3 mg L^–1^) than the drip dispersal (3.6 mg L^–1^), whereas other–P was similar and ranged from 0.3 to 0.6 mg L^–1^ in both drainfields (Table 2). This greater decrease in PO_4_–P in the gravel trench drainfield was attributed to the presence of additional 30.5 cm gravel layer that likely facilitated higher PO_4_–P removal.

**Table 2:** Concentrations of chloride (Cl) and phosphorus forms in the septic tank effluent, background groundwater, and unsaturated and saturated zones of drip dispersal and gravel trench drainfields.

Parameter (n = 64)			Cl	Total P		PO_4_–P		Other–P	
			–––––––––––––––––––––––––––––––mg L^−1^–––––––––––––––––––––––––––––––						
Septic tank effluent		Mean	109	13.0	A	9.8 (75%)^a^	A	3.3 (25%)^a^	A
		Median	94.5	12.8		8.5		1.75	
		Range	67–196	5–35		3.9–26.0		0.2–11.0	
Background groundwater (n = 15)		Mean	26	0.11	FG	0.033 (30%)^a^	E	0.077 (70%)^a^	DEF
		Median	24	0.08		0.01		0.06	
		Range	13–50	0.03–0.11		0.01–0.06		0.10–0.11	
Drip Dispersal	L1 (30.5 cm)	Mean	90	4.20	B	3.60	B	0.60	B
		Range	22–187	0.1–17.0		0.02–16.4		0.01–4.5	
	L2-L3 (61 cm)	Mean	88	0.40	D	0.30	C	0.09	DE
		Range	21–187	0.2–1.3		0.2–1.1		0.01–0.3	
	L4 (106.7 cm)	Mean	85	0.14	FG	0.07	D	0.07	F
		Range	18–172	0.03–0.6		0.02–0.6		0.0–0.2	
	P1-P5 (>300 cm)	Mean	40	0.18	F	0.04	E	0.14	CD
		Range	14–82	0.03–0.6		0.004–0.2		0.009–0.6	
Gravel Trench	L1 (30.5 cm)	Mean	89	1.60	C	1.30	B	0.30	BC
		Range	23–180	0.2–4.5		0.06–3.9		0.01–1.8	
	L2-L3 (61 cm)	Mean	84	0.30	DE	0.18	C	0.12	DE
		Range	30–177	0.09–0.8		0.07–0.5		0.008–0.6	
	L4 (106.7 cm)	Mean	92	0.12	G	0.04	E	0.08	EF
		Range	34–182	0.01–0.7		0.005–0.2		0.003–0.5	
	P1-P5 (>300 cm)	Mean	53	0.30	EF	0.07	E	0.23	CD
		Range	13–119	0.02–1.5		0.002–0.5		0.01–1.3	

^a^ Values in the parentheses are percent of total P

Mean values followed by the same letter in the same column are not significantly different.

ANOVA was performed on the log-transformed data.

Within the drainfields, concentrations of both P forms decreased resulting in <0.14 mg L^–1^ TP at 106.7 cm below the infiltrative surface, which was equivalent to >98% reduction in TP from the STE. A slightly higher PO_4_–P in the drip dispersal (0.07 mg L^–1^) than gravel trench (0.04 mg L^–1^) at 106.7 cm depth is attributed to the smaller infiltrative surface of drip dispersal as compared to the gravel trench system.

Concentrations of PO_4_–P continued to decline from 106.7 cm to >300 cm, with no significant differences observed between groundwater beneath either drainfield and background groundwater (Table 2). Whereas, concentrations of other–P increased by two-three folds from 106.7 cm (0.70–0.80 mg L^–1^) to >300 cm (0.14–0.23 mg L^–1^) in both drainfields. The low concentrations of PO_4_–P found in groundwater could be due to the fact that some PO_4_–P may have been sorbed on the colloidal particles present in solution during storage prior to filtration in the laboratory, thus, contributing to a slightly higher other–P. To avoid this, we recommend filtration in the field during sample collection. This increase could also be due to the chemical reactivity (lack of sorption and mobility) as other–P pool is comprised of organic and colloidal forms and its subsequent accumulation and flushing below drainfields. This suggests that although PO_4_–P is rapidly attenuated in the drainfields, there is a potential for other–P to leach and reach groundwater below septic systems. However, regardless of this increase at >300 cm, there was no significant difference in TP or other–P concentrations between groundwater beneath either drainfield and background groundwater (Table 2).

Overall, our data indicates that effective and efficient removal of P can be accomplished by both drainfield designs as after STE passed through both drainfields and reached groundwater, TP, PO_4_–P, and other–P were reduced by >98%, 99%, and 93–96%, respectively.

**Fig 4:**
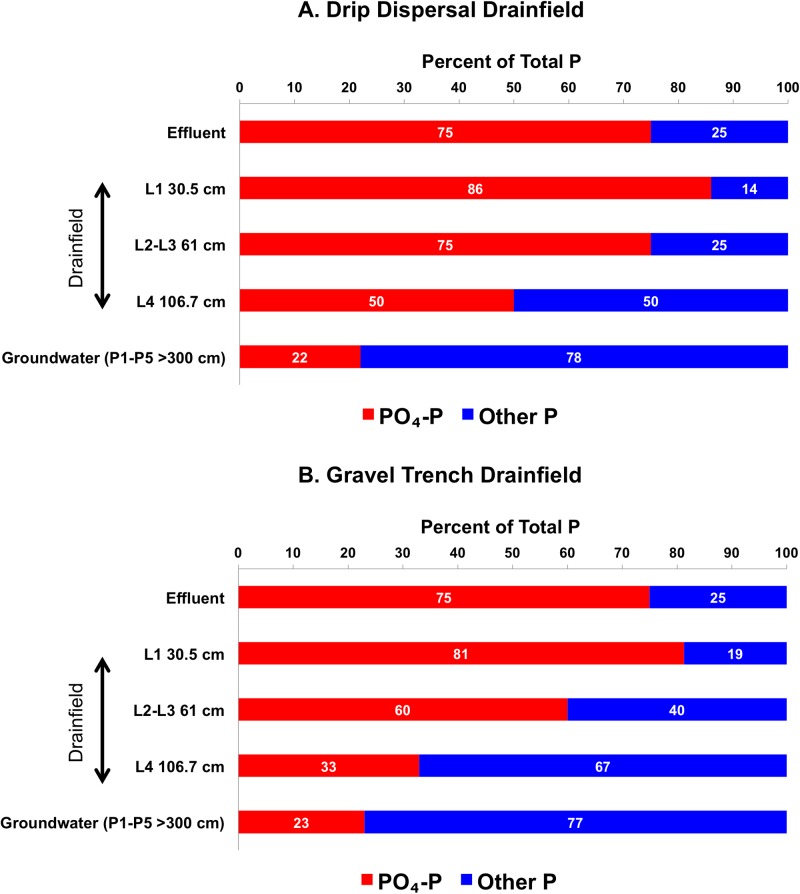
Mean (n = 64) percentages of PO_4_–P and other–P in the effluent, unsaturated zone lysimeters located at 30.5 cm (L1), 61 cm (average of L2 and L3), and 106.7 cm (L4) below the infiltrative surface, and saturated zone piezometers (average of P1 to P5) located at >300 cm depth in the groundwater in the (A) drip dispersal and (B) gravel trench drainfields.

The proportion of PO_4_–P was 75% of TP in the STE, which increased to 81–86% of TP at 30.5 cm and then gradually decreased to <23% of TP at >300 cm below the infiltrative surface in both drainfields (Fig 4). The increase in PO_4_–P accompanied by the decrease in other–P suggests that a part of other–P was mineralized by microbes to PO_4_–P within the first 30.5 cm of the drainfield. Whereas, the decrease in PO_4_–P proportion with depth is attributed to the sorption as well as microbial uptake that immobilized PO_4_–P in and below the drainfields.

The proportion of other–P showed an inverse relationship to PO_4_–P. For example, other–P was 25% of TP in STE, and decreased to 14–19% of TP at 30.5 cm before increasing to 77–78% of TP at >300 cm in both drainfields (Fig 4). The increase in other–P proportion with depth suggests that other–P is more mobile or microbial transformations influenced P proportions in the drainfields. Using the lysimeter studies with dairy slurry and animal manures applications, researchers have demonstrated that organic and particulate P forms dominantly leach from soil to groundwater [20–22]. Thus, our results add to the scientific literature that organic and colloidal forms of P also leach and reach groundwater below septic system drainfields.

### Seasonal Effects on Phosphorus Transport

**Fig 5:**
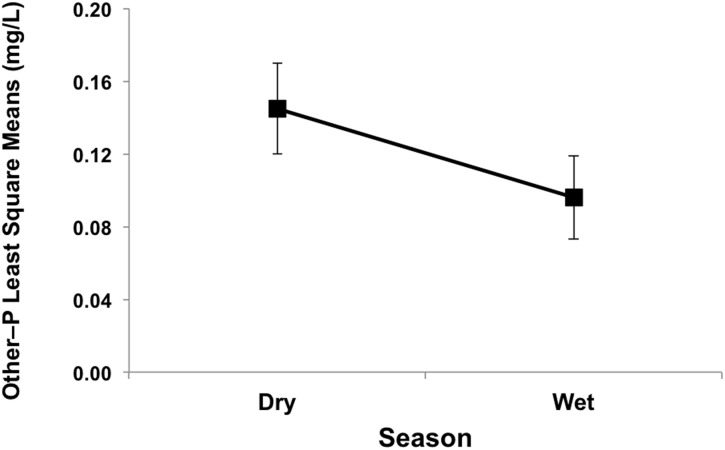
Least square means plot showing overall seasonal difference between dry (October–May) and wet (June–September) seasons for other–P in the drip dispersal and gravel trench septic systems (n = 64).

The results of the two-way ANOVA showed a seasonal effect for other–P and no seasonal effect for PO_4_–P or TP (Table 3). Among P forms, only least square means of other–P with respect to season was significantly higher during the dry than wet season (Fig 5). This was likely caused by the absence of rainfall in the dry season that diluted and reduced other–P concentrations during the wet season. Analysis of the full factorial indicated that there was no combined Season–System/Depth effect for TP, PO_4_–P, or other–P. This indicates that although there was a System/Depth effect and Season effect separately, there was no combined effect.

**Table 3:** Fixed effects test for total P, orthophosphate–P (PO_4_–P), and other–P.

Source	Degrees of Freedom	Probability > F^a^		
		Total P	PO_4_–P	Other–P
Season (wet/dry)	1	0.07	0.19	0.0062*
System/Depth	9	< .0001*	< .0001*	< .0001*
Season*System/Depth	9	0.03	0.11	0.8

^a^ Indicates F-test used in the hypothesis testing.

* Indicates a significant difference at α = 0.05.

## Discussion

### Fate of Phosphorus in Conventional Drainfields

Concentrations of P significantly decreased from STE to 30.5 cm depth in both drainfields, with the gravel trench removing significantly more TP (~20%) than the drip dispersal. The higher TP removal in gravel trench system was likely due to the additional 30.5 cm of gravel layer, which could retain more STE in the gravel pores and provide more side-wall infiltration area than sand layer alone in the drip dispersal system [7]. Total attenuation of TP within the first 30.5 cm was ~90% in the gravel trench and ~70% in the drip dispersal drainfields. This high attenuation was the result the oxidation of effluent in close proximity to the drip line allowing mineral precipitation reactions, which other studies have also found to be a driver of P attenutation [8, 10, 11, 23]. A higher reduction in N was observed within the drip dispersal (49%) as compared with the gravel trench (21%) due to higher gaseous N losses in the drip dispersal drainfield (De & Toor, Ecological Engineering, in review, 2017). The sand used in the drainfields was non-calcareous (Ca 0.1% weight, Table 1), thus, the oxidation of the effluent likely resulted in the development of acidic conditions, which could enhance the precipitation of Fe-P and Al-P minerals and this was more pronounced in the gravel trench as compared to the drip dispersal drainfield.

Our data showed that TP concentrations at 30.5 cm depth increased over time with higher concentrations particularly during the second wet season. This was most evident in the drip dispersal system, which was likely caused by a decrease in the residence time due to the increased rainfall in the second wet season. It is important to note the gravel trench drainfield showed less of an increase over time at this depth, suggesting that the gravel layer may be storing additional STE within the gravel pores, which aided in buffering the system from the seasonal elevated rainfall. For these reasons, the gravel trench system may be more suitable for attenuating P, particularly in Florida where 60–70% of total rainfall in a year occurs during the four-months wet season of June to September [24].

Phosphorus concentrations significantly decreased from 30.5 to 61 cm depth, which contributed to additional TP attenuation of 8.5% in the gravel trench and 28% in the drip dispersal, with cumulative TP reductions of 97% in both systems upto 61 cm depth. Additional 2% TP was attentuated from 61 to 106.7 cm depth, with cumulative TP reduction of approximately 98% in the drip dispersal and 99% in the gravel trench drainfields upto 106.7 cm. Overall, the data indicated that the greatest reductions occurred within the first 61 cm of the drip line and P attenuation slowed down with depth. This high attenutation was likely caused by our newly constructed drainfields with favorable biogeochemical conditions and availability of sorption sites in the surface horizons resulting in limited P transport to lower depths and groundwater. In our earlier study [25], we calculated mass balance of TP by accounting for P inputs and outputs in a drip dispersal system and found that >95% of TP was removed in the first 60 cm depth, which is similar to this study. Further, Mechtensimer & Toor [25] using P sorption isototherms computed that STE application over one-year saturared 18% of P sorption capacity within 60 cm depth of drainfield and that all P sorption sites will be saturated after ~6 years of STE application. This suggest that long-term studies are needed to understand and partition the contribution of septic systems to groundwater P pollution.

### Transport of Phosphorus from Drainfields to Groundwater

Previous research has shown that once P enters the groundwater zone, P attenuation ceases and sorption and precipitation reactions only retard P transport [10, 21, 26]. Our research showed that once P reached the shallow groundwater zone (>300 cm), concentrations of TP were higher than at 61 cm for the gravel trench and 106.7 cm in the drip dispersal (Table 2). This was because other–P was the dominant form at lower depths, which contributed to higher TP in the groundwater. Although in both systems, TP increased from the drainfield to groundwater, the increase was not significantly different from background groundwater and thus we can conclude there was no significant increase in groundwater P concentrations after 18 months of STE application. Total P was reduced by >98% from STE to >300 cm in the groundwater in both systems, although P removal was greater at shallower depths (30.5 and 61 cm) in the gravel trench as compared to drip dispersal system, which may be benefical in areas with elevated groundwater tables or in sensitive karst environments (Figs 2 & 3).

### Seasonal Variability in Phosphorus Species Transport

The two-way ANOVA showed that there was no significant seasonal effect on PO_4_–P or TP, however, other–P was significantly greater during the dry season as compared to the wet season (Fig 5). This increase may be due to the absence of rainfall in the dry season that otherwise diluted other–P concentrations during the wet season. The proportion of other–P increased as it moved through the drainfield. For example, STE had 75% PO_4_–P and 25% other–P, which changed to <25% PO_4_–P and >75% other–P as STE passed through both drainfields and reached groundwater (>300 cm). The decrease in PO_4_–P was a result of the physiochemical nature of PO_4_–P that allows this form to interact with the soil profile and into soil aggerates [21] and increase opportunites for sorption. Further, microbes use inorganic P (i.e., PO_4_–P) from the soil solution for cell development, thus, consuming PO_4_–P [27].

### Conclusions

More than 97% of TP attenuation occurred in the first 61 cm of two newly constructed conventional drainfields (drip dispersal and gravel trench). There was a little difference in the overall performance between the two systems, with the exception of gravel trench drainfield that significantly removed 20% more TP within the first 30.5 cm and had significantly lower PO_4_–P concentrations than the drip dispersal drainfield at 106.7 cm. We observed that other–P concentrations significantly increased in both systems during the dry season and were likely due to the absence of rainfall that diluted other–P during the wet season. The gravel trench drainfield buffered against wet sesaon P increase and did not show overall increasing P trends as was observed in the drip dispersal drainfield. For these reasons, the gravel trench drainfield may be a more reliable design for Florida soils particular in coastal areas where P removal is critical and sandy soils and shallow groundwater limit the unsaturated effluent treatment depth. Overall, the P concentrations in the groundwater below both drainfields systems were not significantly different than background groundwater P concentrations. Thus, we can conclude that after 18 months of STE application, both systems were effective at limiting P transport to groundwater. We suggest that partitioning the contribution of various pollutants from septic systems to groundwater and connected surface waters in coastal areas such as Florida is critical to devise the best drainfield designs that are not only effective at attentuating P, but also other nutrients such as nitrogen and organic contaminants.
